# Pinostrobin Inhibits Nuclear Factor-Kappa B Signaling and Production of Inflammatory Cytokines and Chemokines in Human Macrophages

**DOI:** 10.3390/nu17223589

**Published:** 2025-11-17

**Authors:** Phatcharaporn Budluang, Saranyapin Potikanond, Nitwara Wikan, Wutigri Nimlamool

**Affiliations:** 1 Department of Pharmacology, Faculty of Medicine, Chiang Mai University, Chiang Mai 50200, Thailand; phatcharaporn.bud@cmu.ac.th (P.B.); saranyapin.p@cmu.ac.th (S.P.); 2 Lanna Rice Research Center, Chiang Mai University, Chiang Mai 50200, Thailand

**Keywords:** pinostrobin, THP-1 macrophage, NF-κB, IκB-α, LPS, anti-inflammation, cytokines

## Abstract

**Background/Objectives**: Pinostrobin is a natural flavonoid compound, a specific type of monohydroxyflavanone, found in various plants like fingerroot and honey. It displays various biological activities such as antioxidant, antimicrobial, anti-adaptogenic and anti-inflammatory. However, the anti-inflammatory effect on human macrophages has not yet been investigated. **Methods**: In this study, we reported the effect of pinostrobin on inhibiting production of pro-inflammatory cytokines and chemokines in human THP-1 macrophages exposed to lipopolysaccharide. In addition, the responsible mechanisms of pinostrobin at the molecular level were identified. **Results**: Results from ELISA demonstrated that pinostrobin significantly inhibited production of pro-inflammatory cytokines and chemokines, including IL-6, TNF-α, IL-8, MCP-1/CCL2, and CXCL10/IP10. In addition, data from Western blot analysis revealed that pinostrobin suppressed LPS-induced NF-κB activation, in part through blocking IκB-α phosphorylation and degradation as well as NF-κB phosphorylation and nuclear translocation. Results from immunofluorescence study verified that pinostrobin effectively inhibited IκB-α degradation and NF-κB nuclear translocation. Additionally, pre-treatment of pinostrobin alone retained the existence of IκB-α in the cytoplasm of human macrophages, and this may contribute to the enhanced inhibitory activity of pinostrobin in suppressing NF-κB activation. **Conclusions**: This study provided accumulated evidence that pinostrobin exhibits anti-inflammatory activities, and these properties of pinostrobin in human macrophages stem from the inhibition of the NF-κB signal transduction pathway. Our findings suggest that pinostrobin may be useful for the development of therapeutic treatment of inflammation-related diseases or conditions in which NF-κB is overactive.

## 1. Introduction

Inflammation is widely known to be complicated in pathogenesis, including in inflammation-related diseases and cancer. Several studies showed that inflammation is often associated with the development and progression of cancer [1,2]. However, various stimuli are involved in the inflammatory process, such as imbalance between oxidants and antioxidants that leads to oxidative stress and overproduction of cytokines and chemokines. Prolongation of this process could lead to cellular damage, which initiates chemical chain reactions, including lipid peroxidation and the oxidation of DNA and proteins, resulting in cell dysfunction and cell death [3,4].

Macrophages play both roles in triggering and resolving inflammation, which is a key part of the body’s immune response and inflammation processes. Macrophages can be stimulated into two distinct phenotypes, including pro-inflammatory M1 macrophages and anti-inflammatory M2 macrophages, each contributing to different phases of the immune response [5,6]. The THP-1 cell line exhibits conserved characteristics of human monocytic cells, and it is a proper model widely used for studying immunological responses, including anti-inflammatory properties of drugs or active compounds. Several different stimuli can be used to differentiate THP-1 monocytic cells to macrophages. These include macrophage colony stimulating factor (M-CSF), 1α, 25-dihydroxyvitamin D3 (vD3), and phorbol 12-myristate-13-acetate (PMA) (also known as 12-O-tetradecanoylphorbol 13-acetate (TPA)) [7,8]. TPA is a primary activator for protein kinase C (PKC), which can activate downstream signaling of monocytes to macrophages. After activation with TPA, cells are adherent, are more phagocytic, and have lower rates of proliferation, and they express an increased level of cell surface markers CD11b and CD14 [9,10].

Gram-negative bacteria have lipopolysaccharide (LPS) on their outer membrane, and this component can activate Toll-like receptor 4 (TLR4) in macrophages, leading to the activation of nuclear factor NF-kappa B (NF-κB) signaling [11,12]. Activated NF-κB signaling leads to the production of inflammatory mediators and other inflammation genes, which leads to promotion of pathological inflammation. LPS-stimulated macrophage mediates inflammatory responses by releasing key pro-inflammatory mediators, including interleukin-6 (IL-6), IL-8, and tumor necrosis factor (TNF-α) [13,14]. The binding of LPS and TLR4 triggers a signaling cascade involving the MyD88 and Mal pathway, leading to the activation of the IκB kinase (IKK), which phosphorylates IκB-α and IκB-β, marking them for degradation by the proteasome. With IκB-α released, the NF-κB transcription factor (a complex of p50 and p65) translocates to the nucleus of the cell. NF-κB targets specific response elements and activates the transcription of genes involved in inflammation, including IL-6, IL-8, TNF-α, MCP-1, CXCL10, and others. In addition, the NF-κB signaling can be activated by IL-6, TNF-α, and other molecules involving in inflammation [15,16,17,18]. Thus, inhibiting NF-κB can mitigate inflammation-related diseases [19].

Cytokines such as IL-6 and TNF-α are associated with various diseases, particularly those involving chronic inflammation and systemic autoimmune conditions. According to meta-analysis, it was reported that the overproduction of certain cytokines, such as IL-6, IL-1β, and TNF-α, is a risk factor involved in the development of cardiovascular diseases, neurodegenerative conditions, sarcopenia, and frailties [20,21]. Since IL-6 plays a critical role in initiating and maintaining inflammation that promotes cancer progression, this cytokine is suggested to be a cancer biomarker [22,23,24]. In addition, TNF-α is associated with the pathophysiology of arthritis [25], inflammatory bowel disease (IBD) [26], and some specific types of cancer [27]. Therefore, targeted therapy for TNF-α has been successfully applied to fight some of these diseases even though it can also have some serious adverse effects [28]. Moreover, it is verified that TNF-α polymorphism is a risk factor for several different cancers, including gastric, liver, and breast cancers [29]. Thus, researching new drug therapy to attenuate these pro-inflammatory cytokines could prevent and treat diseases related to inflammation. Additionally, chemokines are involved in attracting and guiding white blood cells to sites of inflammation [30]. For instance, IL-8 attracts immune cells to the site, and this function eventually contributes to a long-lasting accumulation of neutrophils and the generation of local exudation [31]. IL-8 is also involved in cancer development and metastasis through inducing angiogenesis and maintaining cancer stem cells [32]. In addition, IL-8 activates other inflammatory cells, induces the releasing of various chemokines, produces high levels of ROS, increases expression of the integrin CD11b–CD18, enhances endothelial cells adhesion, and modulates histamine release [33,34]. Increased levels of IL-8 have been observed in lung carcinomas and in melanomas [15,35]. MCP-1 (or CCL2) directly recruits peripheral leukocytes to the injured and inflamed areas, such as the atherosclerotic plaque, and thus this chemokine is a key marker of adverse cardiovascular events [36]. Moreover, spontaneous secretion of MCP-1 can induce type 1 diabetes in adults where the cells responsible for insulin production are destroyed [37]. CXCL10 is also a chemokine reported to promote breast cancer cell development and progression [38]. Overproduction of these cytokines and chemokines are associated with inflammation. Thus, finding agents to mitigate this event may be useful to treat inflammation-related diseases.

Pinostrobin or 5-hydroxy-7-methoxyflavanone is a natural flavonoid found in various plants species, including *Cajanus cajan*, *Corymbia torellian*, *Piper ecuadorense*, *Piper hispidum*, *Polygonum ferrugineum*, *Populus tomentosa* Carr, and *Teloxys graveolens*. Pinostrobin is also found in fingerroot (*Boesenbergia rotunda*), which is used as a household herb, herbal medicine, and food ingredient in Southeast Asia. It has been investigated for its potential in traditional medicine and found to have a wide range of biological activities, including antioxidant [39], antimicrobial [40,41], antiadipogenic [42], antitumor [43,44,45], and anti-inflammatory activities [39,46]. In particular, anti-inflammatory effects of pinostrobin in human macrophage cells remain largely unknown. Thus, this study aimed to investigate the anti-inflammatory effects of pinostrobin and elucidated the possible molecular mechanisms underlying its anti-inflammatory activities in activated human macrophages.

## 2. Materials and Methods

### 2.1. Cell Lines and Cell Culture

THP-1 cell line was obtained from ATCC, Number TIB-202 (Manassas, VA, USA), and maintained in complete RPMI (Gibco, New York, NY, USA). To make complete RPMI medium, we added fetal bovine serum (FBS) (Gibco) at the final concentration of 10% (*v*/*v*), penicillin (100 U/mL) and streptomycin (100 μg/mL) (both antibiotics from Gibco), and 2-mercaptoethanol (0.05 mM) (Thermo Fisher Scientific, Waltham, MA, USA). THP-1 cells were cultured in complete medium in a humidified incubator set at 37 °C with 5% CO_2_.

### 2.2. Differentiation of THP-1 Monocytic Cells to Human Macrophage

THP-1 cells are human monocytic cells that can be activated to differentiate to mature macrophages. Since this cell line contains conserved characteristics (innate immune response) of human macrophages in response to inflammatory stimuli (which generally does not exhibit physiological and immunological variation), we thus chose this cell line as a single representative cell line of human macrophages. THP-1 cells were activated in plates with complete RPMI media containing 12-O-tetradecanoylphorbol-13-acetate (TPA) (60 nM) (Thermo Fisher Scientific) for 14 h. Then, the wells were replaced with complete media, and the cells were further cultured for 24 h. The characteristics of adhesive macrophages were confirmed under a light microscope (Olympus, Tokyo, Japan).

### 2.3. Cell Viability Determination of THP-1 Macrophage Treated with Pinostrobin

THP-1 human macrophage cells were cultured in 96-well culture plates at the density of 4 × 10^4^ cells/well. After cells were differentiated to macrophages, pinostrobin (Thermo Fisher Scientific) was added to each well according to varied concentrations of the compound (0–100 µM) then incubated for 24 or 48 h. To assay cell viability, the cells were washed 3 times with PBS, and MTT reagent (Thermo Fisher Scientific) was added to the wells for 1 h in a humidified incubator. The development of formazan color was visualized under a light microscope before cell lysis. Cell lysis was performed by adding 100% DMSO into each well. The color intensity read at 570 nm was determined by a spectrophotometer (BioTek Instruments, Winooski, VT, USA).

### 2.4. Cytokine and Chemokine Production Determinations

Macrophages were pre-treated with or without different concentrations of pinostrobin for 3 h. After 3 h of incubation, 10 ng/mL of LPS was added, and cells were incubated for 24 h. The cell debris was discarded by high-speed centrifugation at low temperature, and the culture supernatants were collected. Then, the culture supernatants were subjected to ELISA for detecting IL-6, TNF-α, IL-8, MCP-1, and CXCL10 following the instruction of the manufacturer (BioLegend, San Diego, CA, USA). The immunoplates were coated with capture antibody (diluted in the coating buffer) overnight at 4 °C. The plates were washed 3 times and blocked with the blocking buffer for 1 h at room temperature (RT). After washing, 100 µL of collected culture supernatants was added to the plates, which were incubated for 2 h at RT. After washing, the detection antibody solution was added to the plates for 1 h, followed by a secondary antibody conjugated with biotin for 1 h. Then the plates were incubated with avidin-horseradish peroxidase (HRP) for 30 min. After washing, TMB substrate was added to the plates, and the development of color was monitored and stopped with a stop solution. The developed color read at 450 and 570 nm was determined by a plate reader (BioTek Instruments, Winooski, VT, USA).

### 2.5. Preparation of Whole-Cell Lysates and Cytoplasmic and Nuclear Extracts

The whole-cell lysates were prepared for measuring the level of proteins in macrophages. Briefly, the cells were pre-treated with pinostrobin (25–100 µM) and stimulated with or without LPS (10 ng/mL) (Thermo Fisher Scientific). Cultured media were removed, and the treated cells were directly extracted by Laemmli SDS sample reagent for 5 min. The whole-cell lysates were collected.

The cytoplasmic and nuclear extracts were prepared by adding lysis buffer A (10 mM HEPES, pH 8.0, 1.5 mM MgCl, 10 mM KCl, 0.5 mM dithiothreitol (DTT), 300 mM sucrose, and 0.1% NP40) containing 1X protease inhibitor on ice for 5 min. The insoluble matter which contained nucleus was removed by centrifugation at 12,000× *g* for 10 min at 4 °C, and the supernatant fraction (cytoplasmic extract) was collected. The pellets of the cell nuclei were washed 1 time with ice-cold PBS (1X) and subjected to centrifugation. Then, PBS was discarded, and the nuclei were lysed with buffer B (20 mM HEPES, pH 8.0, 100 mM NaCl, 100 mM KCl, 0.2 mM EDTA, 0.5 mM DTT, 0.5 mM phenylmethylsulfonyl fluoride (PMSF), and 20% glycerol) before being centrifuged at 12,000× *g* for 10 min at 4 °C. The supernatants (the nuclear fraction) were collected.

### 2.6. Western Blot Analysis

All sample cell lysates (whole-cell lysates, cytoplasmic extract, and nuclear extract) were heated at 95 °C for 5 min. After cooling down, the collecting tubes were short-spun to collect all lysates. The heat-denatured proteins were separated by 10% gel SDS-PAGE and blotted onto PVDF membrane (Thermo Fisher Scientific). Membranes were incubated with 3% BSA in tween-TBS for 1 h and incubated with specific antibodies of interest at 4 °C overnight. All antibodies were purchased from Cell Signaling Technology (Danvers, MA, USA). In this study, we used antibodies against beta actin (Cat No. 3700), phospho (Ser536)-NF-κB (Cat No. 3033), total NF-κB (Cat No. 8242), phospho (Ser32)-IκB (Cat No. 2859), total IκB (Cat No. 4814), LaminA/C (Cat No. 2023), and tubulin (Cat No. 3873). After washing, secondary antibodies were added, and the membranes were incubated at RT for 2 h. All secondary antibodies were purchased from LI–COR Biosciences (Lincoln, NE, USA). Secondary antibodies were anti-mouse IgG conjugated with IRDye 800CW (Cat No. 926-32210) and anti-rabbit IgG conjugated with IRDye 680RT (Cat No.926-68071). Western blot band intensity was determined by an Odyssey^®^ CLx Imaging System (LI–COR Biosciences) membrane scanner. Densitometry analyzing the area under the curve of each immunoreactive band was performed by ImageJ software version 1.51j8, supported by NIH (Bethesda, MD, USA). Quantification of the phosphorylation status of each protein was performed by normalizing the phosphorylated form with its total form. Notably, due to the interference of the reducing agents present in the lysis buffer, protein assay and loading normalization were unfeasible. Therefore, the immunoreactive band intensity of the total form of a specific protein was mainly normalized with that of cytoskeletal proteins (actin, tubulin, or Lamin). However, these housekeeping proteins and total loaded proteins may not show the best proportionality, and this was considered a limitation as reported by a previous study [47].

### 2.7. Immunofluorescence Assay

Differentiated THP-1 macrophages were cultured on round glass coverslips (Thermo Fisher Scientific) inserted in each well of 24-well plates (Corning, Kennebunk, ME, USA). Then, culture supernatants were removed, and 4% formaldehyde solution was added to each well to fix cells for 15 min at RT. After washing 3 times, the fixed cells were incubated with permeabilizing solution (0.3% TrironX-100/PBS) for 5 min at RT. Cells were incubated with 1% BSA/TBST for 1 h at RT to block non-specific background. Then, cells were co-incubated with antibodies against IκB and NF-κB for 24 h at 4 °C. These antibodies were the same as those used in Western blot analysis, but the dilution was prepared following the company suggestions for immunofluorescence study application. The next day, cells were incubated with a mixed solution containing goat anti-rabbit IgG conjugated with Alexa555 and goat anti-mouse IgG conjugated with Alexa488 (both from Thermo Fisher Scientific) and DAPI (Cat No. 4083) (Cell Signaling Technology) for detecting the cell nuclei. The coverslips were mounted onto a fluorescent microscope glass slide using an anti-fade medium (Leica Microsystems Ltd., Wetzlar, Germany). Visualization and image analysis were performed by a fluorescent microscope (A Leica DMi8 Thunder Imager 3D Assay) equipped with LAS X image-processing software version 3.8.1 (Leica Microsystems Ltd.).

### 2.8. Statistical Analysis

Each assay was performed in at least 3 individual experiments. Experimental results were quantified and arranged by using version 9.0.0 of GraphPad Prism software by GraphPad SoftwareInc (San Diego, CA, USA). All quantified results were shown as the mean ± SD. The results were subjected to one-way ANOVA (followed by Tukey’s multiple comparison test). Determination of statistical significance was determined if the *p* value was less than 0.05 (<0.05).

## 3. Results

### 3.1. Effect of Pinostrobin on Cell Viability of Macrophages

**Figure 1:**
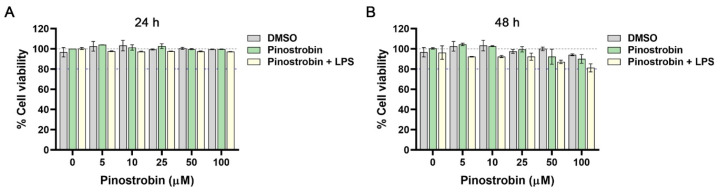
Effect of pinostrobin on cell viability of THP-1 human macrophages. Cells were treated with pinostrobin at the concentration range of 0–100 µM or pinostrobin with the presence of 10 ng/mL LPS or DMSO as a vehicle control for 24 h (**A**) and 48 h (**B**) prior to the viability assay. Gray line (dashed) indicates 100% of cell viability. The blue line indicates 80% of cell viability. The results are obtained from 3 biological replicates and expressed as mean ± SD.

To assess the cytotoxicity, THP-1 macrophages were treated with different concentrations of pinostrobin (0–100 µM) for 24–48 h. The reduction of cell viability (lower than 80%) was considered toxic to the cells. The result showed that at 24 h, all conditions of treatment, including DMSO (as a vehicle control), pinostrobin alone (0–100 µM), and pinostrobin (0–100 µM) with the presence of LPS (10 ng/mL), had no toxicity to the cells as observed by MTT assay (Figure 1A). At 48 h, even though it showed a slight reduction in cell viability, pinostrobin at concentrations up to 100 µM was not toxic to the cells since the cell viability was still maintained above 80% (Figure 1B).

### 3.2. Effect of Pinostrobin on LPS-Induced Cytokine and Chemokine Production in Human Macrophages

**Figure 2:**
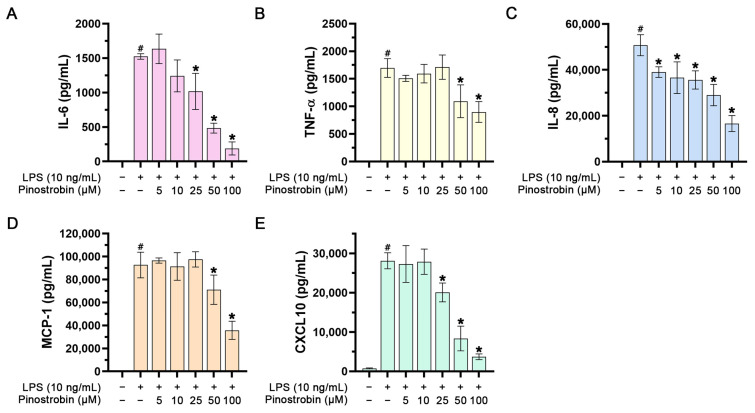
Effect of pinostrobin on IL-6, IL-8, TNF-α, MCP-1, and CXCL10 production and release in the culture supernatants of LPS-induced THP-1 human macrophages. The levels of IL-6 (**A**), TNF-α (**B**), IL-8 (**C**), MCP-1 (**D**), and CXCL10 (**E**) in the media of pinostrobin-treated cells stimulated with 10 ng/mL of LPS for 24 h evaluated by ELISA. The results are obtained from 3 biological replicates and expressed as mean ± SD. # *p* < 0.05 (compared with the control group without any treatment). * *p* < 0.05 (compared with LPS-treated cells).

To investigate whether pinostrobin could negatively regulate inflammatory cytokines, we measured the level of inflammatory cytokines, including IL-6 and TNF-α, and the level of the chemokines, including IL-8, MCP-1, and CXCL10, in the culture supernatants of treated macrophages collected at 24 h. As shown in Figure 2A–E, the culture supernatants collected from macrophages without any treatment had no or very low detectable levels of IL-6, TNF-α, IL-8, MCP-1, and CXCL10. Stimulation of macrophages with LPS for 24 h increased the levels of IL-6, IL-8, TNF-α, MCP-1, and CXCL10 to approximately 1500 pg/mL, 1700 pg/mL, 50,000 pg/mL, 90,000 pg/mL, and 28,000 pg/mL, respectively. However, pre-treatment of cells with pinostrobin for 3 h prior to LPS stimulation could significantly reduce the levels of IL-6, TNF-α, IL-8, MCP-1, and CXCL10 in a dose-dependent manner.

### 3.3. Effect of Pinostrobin on NF-κB Activation in LPS-Induced Human Macrophages at Various Time Points

**Figure 3:**
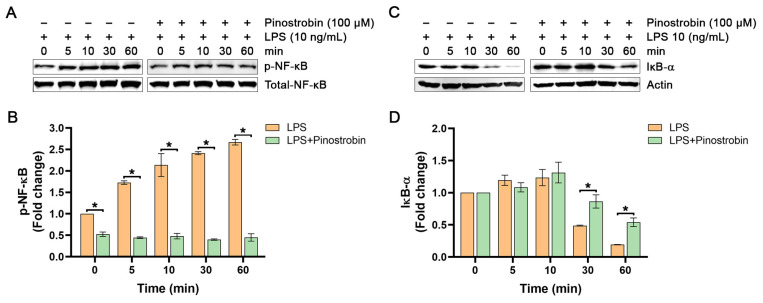
Western blot analysis examining the effects of pinostrobin on the phosphorylation of NF-κB and the total amount of IκB-α in THP-1 human macrophages after LPS exposure at various time points. (**A**) Western blot analysis showing immunoreactive bands of p-NF-κB. (**B**) Quantitative analysis of p-NF-κB immunoreactive bands. (**C**) Western blot analysis showing immunoreactive bands of IκB-α protein. (**D**) Quantitative analysis of IκB-α immunoreactive bands. Total NF-κB and actin were used as internal controls. The results are obtained from 3 biological replicates and expressed as mean ± SD. * *p* < 0.05 is considered statistically significant (compared with the LPS-treated cells at each time point).

Based on the previous experiments showing that pinostrobin inhibited the production of cytokines (IL-6 and TNF-α) and chemokines (IL-8, MCP-1, and CXCL10), this suggests that pinostrobin may suppress the upstream signal transduction pathway that regulates the production of these pro-inflammatory mediators. It is well established that the expression of these inflammatory cytokines and chemokines in response to LPS exposure is modulated by a crucial molecular player, NF-κB. Thus, we defined the possible molecular mechanism of action of pinostrobin by focusing on NF-κB. In addition, an inhibitor of kappa B-α (IκB-α) was also investigated, as it is a key factor in regulating the activity of NF-κB. We first observed the phosphorylation status of NF-κB at the serine 536 residue (p-NF-κB) and the level of IκB-α in LPS-induced macrophages with or without pinostrobin at various time points. The data showed that LPS induced the phosphorylation of NF-κB over the course of 60 min, with the peak of phosphorylation showing an approximately 2.5-fold increase at around 30–60 min (Figure 3A,B). However, pre-treatment of cells with pinostrobin (100 µM) for 3 h prior to LPS stimulation significantly reduced NF-κB phosphorylation at all time points (Figure 3A,B). Notably, the level of the unphosphorylated form of NF-κB (total NF-κB) of all treatment groups was not different upon LPS stimulation. As it is well established that LPS exposure eventually triggers IκB-α degradation to release NF-κB, we thus detected and observed that the level of IκB-α started to decrease at 30 min, and the maximal degradation was seen at 60 min in response to LPS exposure (Figure 3C,D). Interestingly, pinostrobin significantly prevented LPS-induced IκB-α degradation at these two time points (Figure 3C,D).

**Figure 4:**
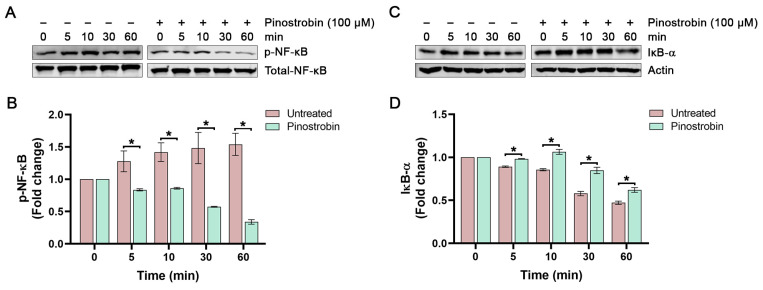
Western blot analysis examining the effects of pinostrobin on the phosphorylation of NF-κB and the total amount of IκB-α in THP-1 human macrophages after beng pre-treated with pinostrobin for 3 h and then harvested at various time points. (**A**) Western blot analysis showing immunoreactive bands of p-NF-κB. (**B**) Quantitative analysis of p-NF-κB immunoreactive bands. (**C**) Western blot analysis showing immunoreactive bands of IκB-α. (**D**) Quantitative analysis of IκB-α immunoreactive bands. Total NF-κB and actin were used as internal controls. The results are obtained from 3 biological replicates and expressed as mean ± SD. * *p* < 0.05 is considered statistically significant (compared with the untreated cells at each time point).

Next, we examined the effect of pinostrobin alone on p-NF-κB and IκB-α degradation level in cells without LPS stimulation. As shown in Figure 4A,B, the basal level of p-NF-κB slightly increased over time, but pinostrobin could significantly decrease this intracellular NF-κB phosphorylation at all detected time points without affecting the total form of NF-κB. As expected, pinostrobin treatment significantly prevented IκB-α degradation at all time points (Figure 4C,D).

**Figure 5:**
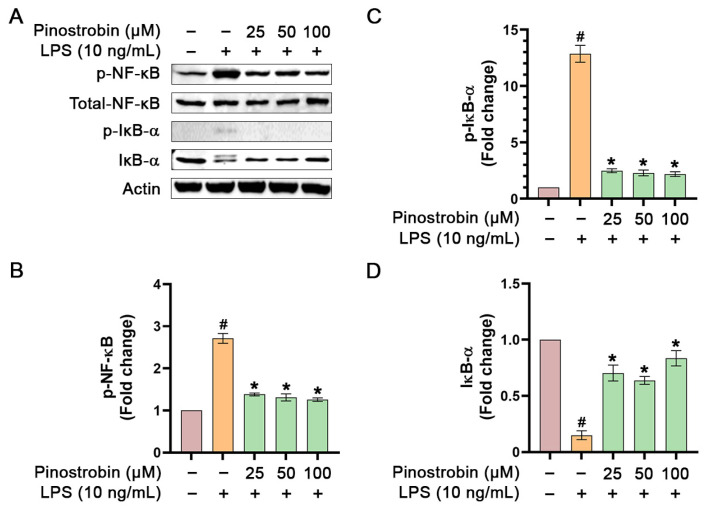
Western blot analysis examining the effects of pinostrobin at various concentrations on the phosphorylation of NF-κB and IκB-α in THP-1 human macrophages exposed to LPS for 1 h. (**A**) Western blot analysis showing immunoreactive bands of p-NF-κB, p-IκB-α, total NF-κB, and total IκB-α. (**B**) Quantitative analysis of p-NF-κB immunoreactive bands, (**C**) p-IκB-α immunoreactive bands, and (**D**) total IκB-α. Total NF-κB and actin were used as internal controls. The results are obtained from 3 biological replicates and expressed as mean ± SD. # *p* < 0.05 is considered statistically significant (compared with the untreated cells). * *p* < 0.05 is considered statistically significant (compared with LPS-treated cells).

To further investigate whether the inhibitory activity of pinostrobin on NF-κB signaling can still be observed when used at lower concentrations, we pre-treated THP-1 human macrophage with pinostrobin at 25, 50, and 100 µM for 3 h and stimulated the cells with LPS for 1 h. Results demonstrated that treatment of THP-1 macrophages with LPS strongly increased the phosphorylation of NF-κB, with an approximately 2.5-fold increase, but pinostrobin at all concentrations could significantly reduce the LPS-induced phosphorylation of NF-κB without affecting the total form of NF-κB (Figure 5A,B). Moreover, LPS stimulation resulted in a strong, approximately 13-fold increase in IκB-α phosphorylation at Ser32, but this LPS-stimulated IκB-α phosphorylation was significantly prevented by pinostrobin at all concentrations (Figure 5A,C). As it is well established that phosphorylation at Ser32 of IκB-α upon LPS stimulation serves as a recognition site for proteasome-mediated degradation, we thus detected the total form of IκB-α and found that LPS stimulation induced the degradation of IκB-α. However, pre-treatment with pinostrobin at all selected concentrations significantly inhibited LPS-induced IκB-α degradation (Figure 5A,D).

**Figure 6:**
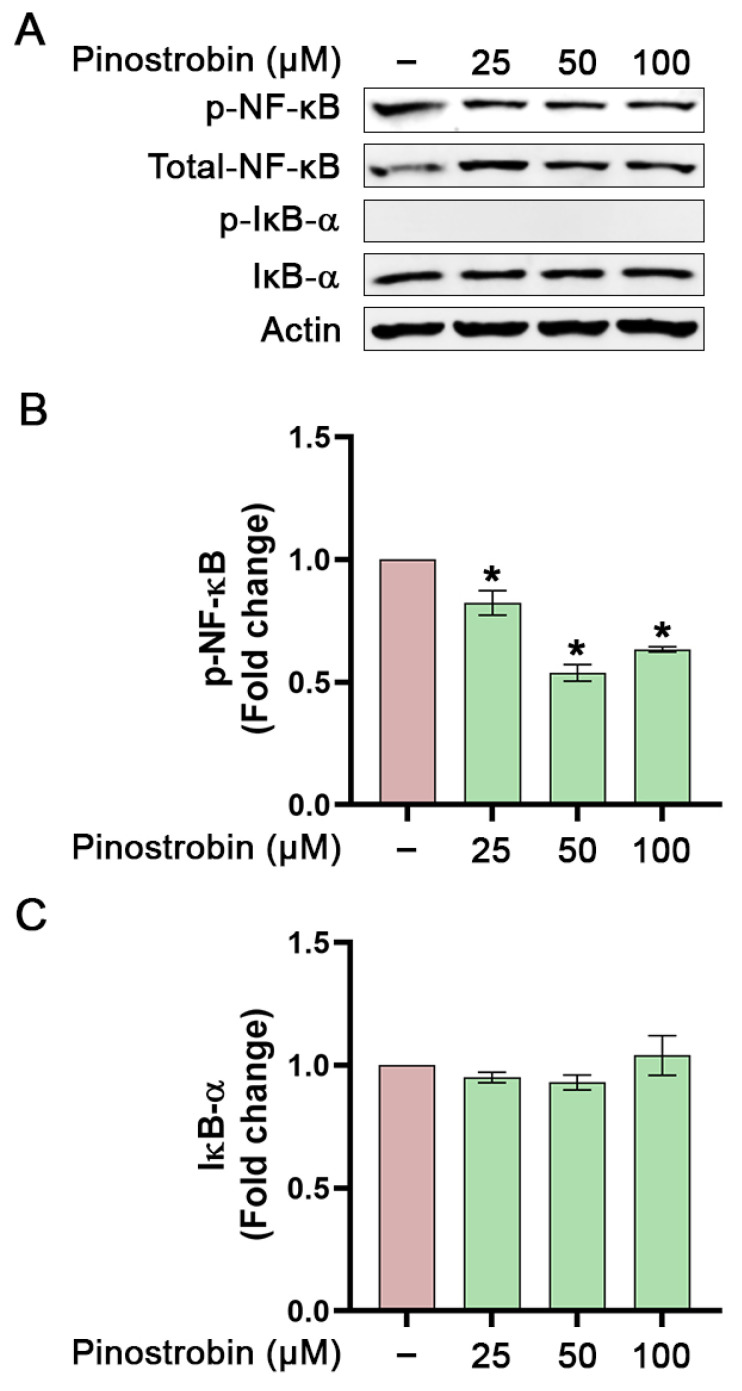
Western blot analysis examining the effects of pinostrobin on the activation of NF-κB and IκB-α in THP-1 human macrophages after being pre-treated with pinostrobin alone and left for 1 h further at various concentrations. (**A**) Western blot analysis showing immunoreactive bands of p-NF-κB, p-IκB-α, total NF-κB, and total IκB-α. (**B**) Quantitative analysis of p-NF-κB immunoreactive bands. (**C**) Quantitative analysis of total IκB-α immunoreactive bands. Total NF-κB and actin were used as internal controls. The results are obtained from 3 biological replicates and expressed as mean ± SD. * *p* < 0.05 is considered statistically significant (compared with the untreated cells).

Without LPS stimulation, data confirmed that pinostrobin (at all concentrations) significantly reduced the basal level of NF-κB phosphorylation (Figure 6A,B). However, in the condition where LPS was absent, the phosphorylation of IκB-α was not induced, and pinostrobin treatment did not affect the level of total IκB-α (Figure 6A,C).

### 3.4. Inhibitory Effect of Pinostrobin on LPS-Induced NF-κB Nuclear Translocation

**Figure 7:**
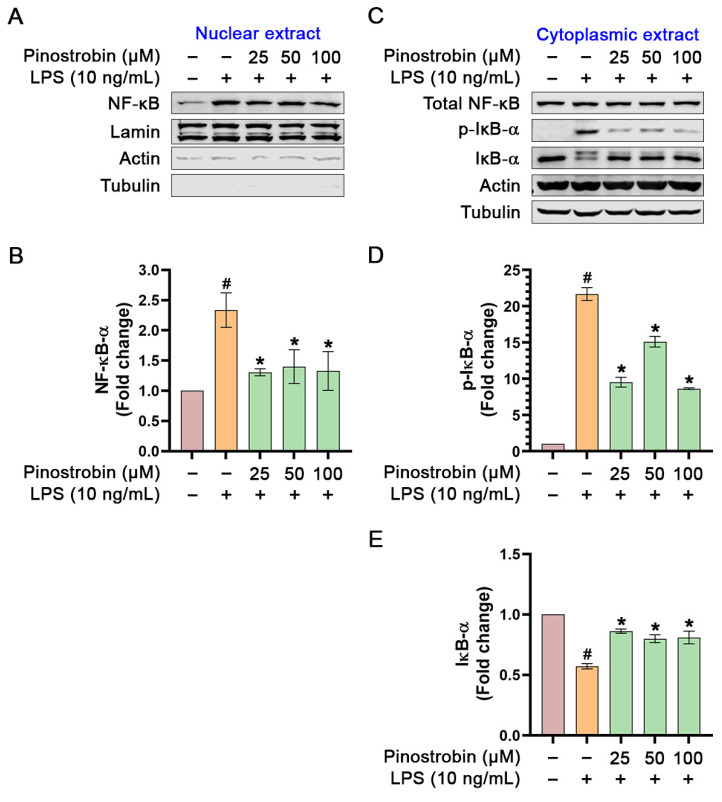
Western blot analysis examining the effects of pinostrobin on the phosphorylation status of NF-κB and IκB-α in the nuclear and cytoplasmic extracts of THP-1 human macrophages after being pre-treated with pinostrobin then exposed to LPS for 1 h. (**A**) Western blot analysis showing immunoreactive bands of total NF-κB in the nuclear extract. (**B**) Quantitative analysis of NF-κB immunoreactive bands in nuclear extract. (**C**) Western blot analysis showing immunoreactive bands of total NF-κB, total IκB-α, and p-IκB-α in the cytoplasmic extract. (**D**) Quantitative analysis of p-IκB-α immunoreactive bands in the cytoplasmic extract. (**E**) Quantitative analysis of total IκB-α immunoreactive bands in the cytoplasmic extract. Lamin, actin, and tubulin were used as internal controls for nuclear extracts, and actin and tubulin were used as internal controls for cytoplasmic extracts. The results are obtained from 3 biological replicates and expressed as mean ± SD. # *p* < 0.05 is considered statistically significant (compared with the untreated cells). The exposure time for actin and tubulin in the nuclear and cytoplasmic extracts was 1 min. * *p* < 0.05 is considered statistically significant (compared with LPS-treated cells).

NF-κB translocation to the nucleus in response to the extracellular stimuli is a critical step in its activation and function as a transcription factor which initiates its downstream gene expressions. To examine whether pinostrobin has effects on NF-κB activation and translocation, cytoplasmic and nucleus fractions of LPS-induced THP-1 macrophages were prepared, and Western blotting was performed to monitor NF-κB translocation and IκB-α phosphorylation and degradation. Results from Western blot analysis demonstrated that the amount of NF-κB in the nuclear extract of LPS-stimulated cells increased approximately 1.5-fold in comparison to that of the untreated cells (Figure 7A,B). Pre-treatment of THP-1 macrophages with pinostrobin significantly decreased LPS-mediated NF-κB translocation to nucleus when compared with LPS stimulation control (Figure 7A,B). A nuclear-residing protin, Lamin, was used as an internal control and for normalization. Moreover, we confirmed the purity of the nuclear extract by detecting actin and tubulin and found that tubulin was totally negative, but faint bands of actin were detected (Figure 7A). Actin can be found in the nucleus since this cytoskeletal protein plays crucial roles in many nuclear processes, including chromatin remodeling, transcription, and DNA repair. In addition, we detected the existence of total NF-κB and total IκB-α (along with its phosphorylated form) in the cytoplasmic extract of the cells upon pinostrobin treatment and LPS stimulation. The result showed that the LPS-stimulation group significantly induced IκB-α phosphorylation and degradation when compared with the untreated group (Figure 7C–E). Nevertheless, pre-treatment with pinostrobin at all concentrations could significantly prevent LPS-induced phosphorylation and degradation of IκB-α (Figure 7C–E). The level of total NF-κB was also detected in the cytoplasmic extract, and it was found to be approximately equal in all groups (Figure 7A).

**Figure 8:**
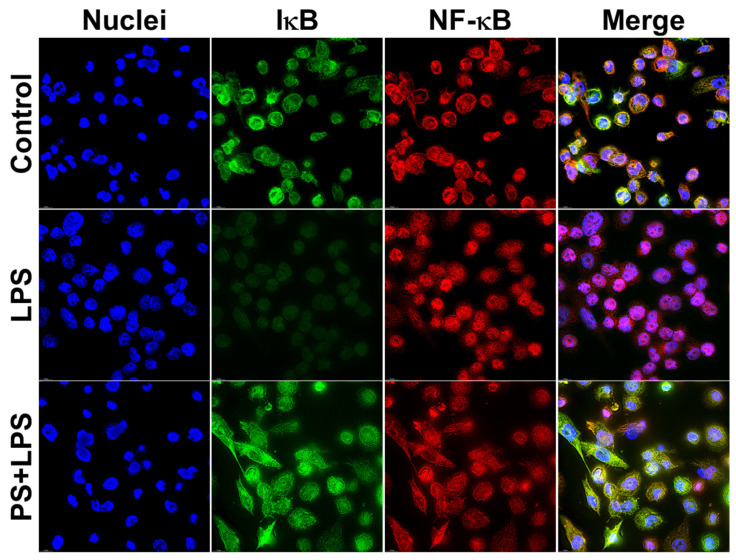
Immunofluorescence study examining the effects of NF-κB on the presence and localization of NF-κB and IκB-α upon LPS stimulation. LPS-induced THP-1 human macrophages for 60 min. Green is IκB-α and red is NF-κB. Cells were counterstained for nuclei (blue) of cells with DAPI. Scale bar = 500 μm, 100× magnification.

Moreover, these observations by Western blot analysis were verified by immunofluorescence study, which clearly indicated that pinostrobin strongly inhibited the localization of NF-κB into the nucleus upon LPS stimulation (Figure 8). The immunofluorescence study data revealed that LPS stimulated the accumulation of NF-κB in the nucleus and induced the degradation of IκB-α in the cytoplasm compared with the negative control. However, pinostrobin treatment retained IκB-α in the cytoplasmic compartment of the LPS-stimulated cells and reduced accumulation of NF-κB in the cell nuclei (Figure 8).

## 4. Discussion

The overproduction and prolonged secretion of pro-inflammatory mediators from macrophages contribute to the pathophysiology of many conditions and diseases, including cancer [48,49], atherosclerosis [50,51,52], dermatitis [53,54], and arthritis [55,56]. Hence, good management of inflammation may alleviate the severity of inflammation-related diseases. Searching for agents that decrease the level of inflammatory cytokines and chemokines may result in the development of novel effective therapeutic strategies for relieving inflammation-associated diseases. In this study, we reported that pinostrobin exhibited anti-inflammatory properties in LPS-induced human THP-1 macrophages.

Data from MTT cell viability assay indicated that pinostrobin (up to 100 µM) was relatively safe, as it showed no toxic effect on THP-1 macrophage cells (even with the presence of LPS) over the course of 48 h. These results are in line with previous studies reporting that pinostrobin at a similar concentration range is also non-toxic in other cell lines. For example, 3T3-L1 preadipocytes (a mouse cell line) were unaffected by pinostrobin up to 100 µM after 48 h of incubation [42]. Moreover, Raw264.7 mouse macrophages could tolerate pinostrobin up to 40 µM for 48 h [41], while peripheral blood mononuclear cells (PBMCs) were unaffected by pinostrobin at a concentration up to 300 µM at 48 h [43]. Additionally, certain cancer cell lines such as melanoma cell (B16F10) were not affected by pinostrobin up to 200 µM for 72 h [57]. In animal studies, pinostrobin at a concentration of 1000 mg/kg showed no sign of toxicity to animals with acute dermatitis condition [58]. Altogether, pinostrobin has been verified to be potentially safe, and it may provide broad therapeutic windows. We next evaluated functional tests related to anti-inflammatory activity of pinostrobin and defined its possible molecular mechanism of action in THP-1 cells.

Since the production and secretion of inflammatory mediators including cytokines and chemokines are the key markers of inflammation, we thus assessed the activity of pinostrobin in LPS-stimulated human macrophages by using ELISA. Results showed that pinostrobin markedly suppressed IL-6, TNF-α, and chemokines (IL-8, MCP-1, CXCL10). These findings indicate that pinostrobin possesses anti-inflammatory activities, and this compound may be a candidate for managing inflammation-related diseases. To ensure how pinostrobin acts at the molecular level, we further investigated the molecular mechanism of pinostrobin. Based on our findings that pinostrobin reduced the production of cytokines and chemokines, we thus focused on identifying molecular mechanisms of pinostrobin in suppressing cytokine and chemokine production. Since NF-κB is a major transcription factor that modulates the production of inflammatory molecules, including IL-6, TNF-α, IL-8, MCP-1, and CXCL10 [13,17,59,60], we also defined the responsible mechanism of action of pinostrobin by focusing mainly on the NF-κB pathway. NF-κB is a complex of p50/p65 heterodimer, which binds to IκB kinase (IKK complex) and IκB-α [13]. For stimulating the NF-κB cascade, LPS is commonly used to activate the NF-κB cascade [14,61]. LPS binds and activates TLR4 receptor, resulting in recruitment of myeloid differentiation primary response gene 88 (MyD88). Then, MyD88 recruits adapter proteins and E3 ligases, such as tumor necrosis factor receptor-associated factor 6 (TRAF6) and downstream interleukin-1 receptor-associated kinases (IRAKs). The activated IRAKs further causes sequential phosphorylation of downstream target proteins that consequently results in IκB-α degradation and NF-κB nucleus translocation [11,12,13]. NF-κB then binds to its promoter regions of various inflammatory genes in the cell nucleus [62]. Thus, we examined the effect of pinostrobin on the phosphorylation and degradation of IκB-α. Furthermore, we determined the phosphorylation status and the translocation of NF-κB into the nucleus upon LPS stimulation. We found that pinostrobin inhibited phosphorylation of IκB-α and prevented the degradation of IκB-α upon LPS stimulation. In addition, pinostrobin strongly inhibited NF-κB phosphorylation. These observations suggest that pinostrobin helps stabilize IκB-α from degradation and that NF-κB is sequestered in its inactive status. To ensure this statement, we monitored the relocalization of NF-κB into the nucleus upon LPS treatment with the presence of pinostrobin. As anticipated, pinostrobin prevented LPS-induced nuclear translocation of NF-κB. Additionally, exposing cells with pinostrobin alone without LPS stimulation showed that the basal level of IκB-α in the cell was increased over the course of incubation. This phenomenon suggests that pinostrobin can retain the existence or delay the normal physiological turnover of IκB-α. Altogether, this study discovered that pinostrobin causes IκB-α accumulation in the cytosol by inhibiting the phosphorylation at serine 32 of this molecule, and this event prevents the relocalization of NF-κB into the nucleus of the cell. This phenomenon may explain its inhibitory actions on the production of human inflammatory markers in response to LPS stimulation. Our findings are consistent with a previous study where rodent cells (RAW 264.7) were utilized to show anti-inflammatory effects of pinostrobin. The previous study identified the cellular target molecules of pinostrobin by using functional tests and molecular docking simulation. The study suggested that pinostrobin might directly bind to the MD2 and TLR4 components of the receptor complex. This binding inhibits NF-κB activation in response to inflammatory stimuli such as lipopolysaccharide (LPS) by hindering LPS binding to the TLR4/MD2 complex [63]. Therefore, pinocembrin may possibly exert this conserved mechanism of action in many cell types including our activated human macrophage model. Other natural compounds have also been reported to display anti-inflammatory effects through modulating NF-κB signaling. For instance, our previous study reported that panduratin A (a compound predominantly found in the fingerroot plant) could inhibit NF-κB phosphorylation and nuclear translocation in microvascular endothelial cells exposed to TNF-α, and that suppressed endothelial activation [64].

Even though we provided promising evidence that pinostrobin can negatively modulate the NF-κB/IκB pathway in activated human macrophages, which may draw attention as an attractive therapeutic target for drug discovery, it needs further tremendous investigation in both animals and humans, especially with regard to safety and efficacy, to be certain that pinostrobin is a safe and effective agent for use in the clinical setting. This statement can be supported by the recent challenges of several different IKKβ inhibitors with well-defined mechanisms of action that target the NF-κB signaling, which still lack clinical success. Specifically, concerns about the safety of IKKβ inhibitors, especially inhibition of systemic inhibition of IKKβ, limit the clinical approval of IKKβ inhibitors [65].

## 5. Conclusions

**Figure 9:**
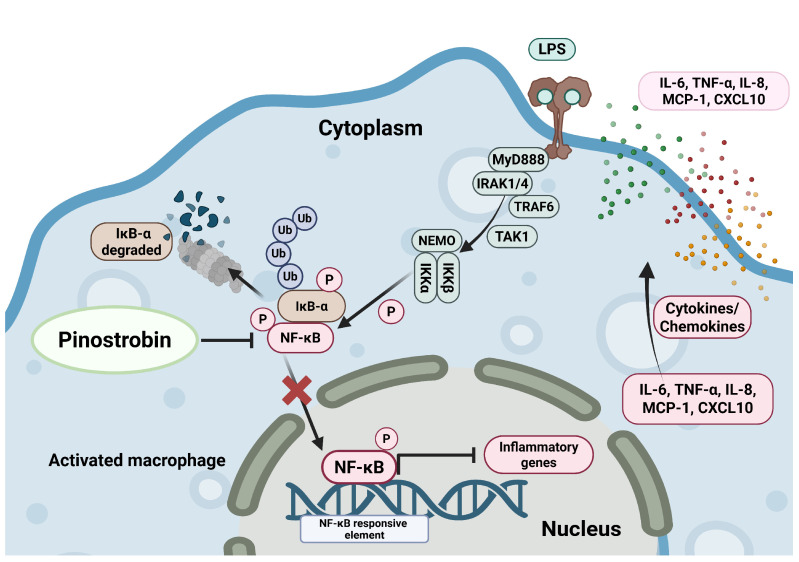
Proposed mechanism of pinostrobin inhibiting LPS-induced inflammation in human macrophage THP-1. Pinostrobin inhibits NF-κB activation and nuclear translocation resulting in decreased production of inflammatory cytokines and chemokines. The schematic illustration was created by BioRender.com.

Our current investigation reveals the anti-inflammatory effects of pinostrobin on the NF-κB signal transduction pathway in human macrophage. The proposed mechanism of action of pinostrobin is illustrated in Figure 9. In particular, pinostrobin reduces the production and secretion of major inflammatory cytokines and chemokines, including IL-6, TNF-α, IL-8, MCP-1, and CXCL10, at least in part through preventing LPS-induced IκB-α phosphorylation and degradation as well as by suppressing NF-κB phosphorylation and nuclear translocation. This study provides accumulated evidence supporting promising benefits of pinostrobin in mitigating inflammation, showing that pinostrobin may be considered for possible use as a potential treatment for inflammatory-related diseases.

## Data Availability

Data are contained within the article.
